# Salvage Surgery Following Definitive Chemoradiotherapy and Immune Checkpoint Inhibitor Therapy for Locally Advanced Thymic Carcinoma

**DOI:** 10.1093/icvts/ivag031

**Published:** 2026-02-10

**Authors:** Ryosuke Tokuda, Satoshi Ikebe, Saki Nishimura-Hanafusa, Masayoshi Inoue

**Affiliations:** Department of General Thoracic Surgery, Fukuchiyama City Hospital, 231 Atsunaka-machi, Fukuchiyama, Kyoto, 620-8505, Japan; Department of General Thoracic Surgery, Fukuchiyama City Hospital, 231 Atsunaka-machi, Fukuchiyama, Kyoto, 620-8505, Japan; Department of General Thoracic Surgery, Fukuchiyama City Hospital, 231 Atsunaka-machi, Fukuchiyama, Kyoto, 620-8505, Japan; Division of Thoracic Surgery, Department of Surgery, Graduate School of Medical Science, Kyoto Prefectural University of Medicine, 465 Kajii-cho, Kamigyo-ku, Kyoto, 602-8566, Japan

**Keywords:** immune checkpoint inhibitors, programmed cell death-ligand 1, salvage surgery, thymic carcinoma, thymic epithelial tumour

## Abstract

Surgical resection improves prognosis for thymic carcinoma. Recent phase II trials have indicated the efficacy of immune checkpoint inhibitor therapy in unresectable disease. Here, we report a case of salvage surgery following immune checkpoint inhibitor therapy for an initially unresectable, locally advanced thymic carcinoma in a 67-year-old woman. Computed tomography revealed an anterior mediastinal mass and enlarged anterior mediastinal lymph nodes, diagnosed as an unresectable thymic epithelial tumour, due to suspected invasion into the left main pulmonary artery. Thoracoscopic biopsy confirmed the diagnosis of squamous cell carcinoma with high programmed cell death-ligand 1 expression (90%-100%). Following definitive chemoradiotherapy, the patient received durvalumab, which reduced the primary tumour size and resolved lymphadenopathy, without immune-related adverse events. Salvage surgery was performed without invasion of the great vessels. The patient remained disease-free at 2 years postoperatively. Salvage surgery following immune checkpoint inhibitor therapy may be a viable treatment option for thymic carcinoma with high programmed cell death-ligand 1 expression.

## INTRODUCTION

Thymic carcinoma (TC) is a rare, aggressive malignancy. Complete resection offers a favourable prognosis. However, robust evidence regarding the role of salvage surgery is lacking.^1^ For unresectable TC, treatment options have been limited. Several phase II trials have demonstrated the efficacy of immune checkpoint inhibitor (ICI) therapy.

Here, we report a case of salvage surgery following ICI therapy in a patient with initially unresectable, locally advanced TC.

## CASE REPORT

**Figure 1.**
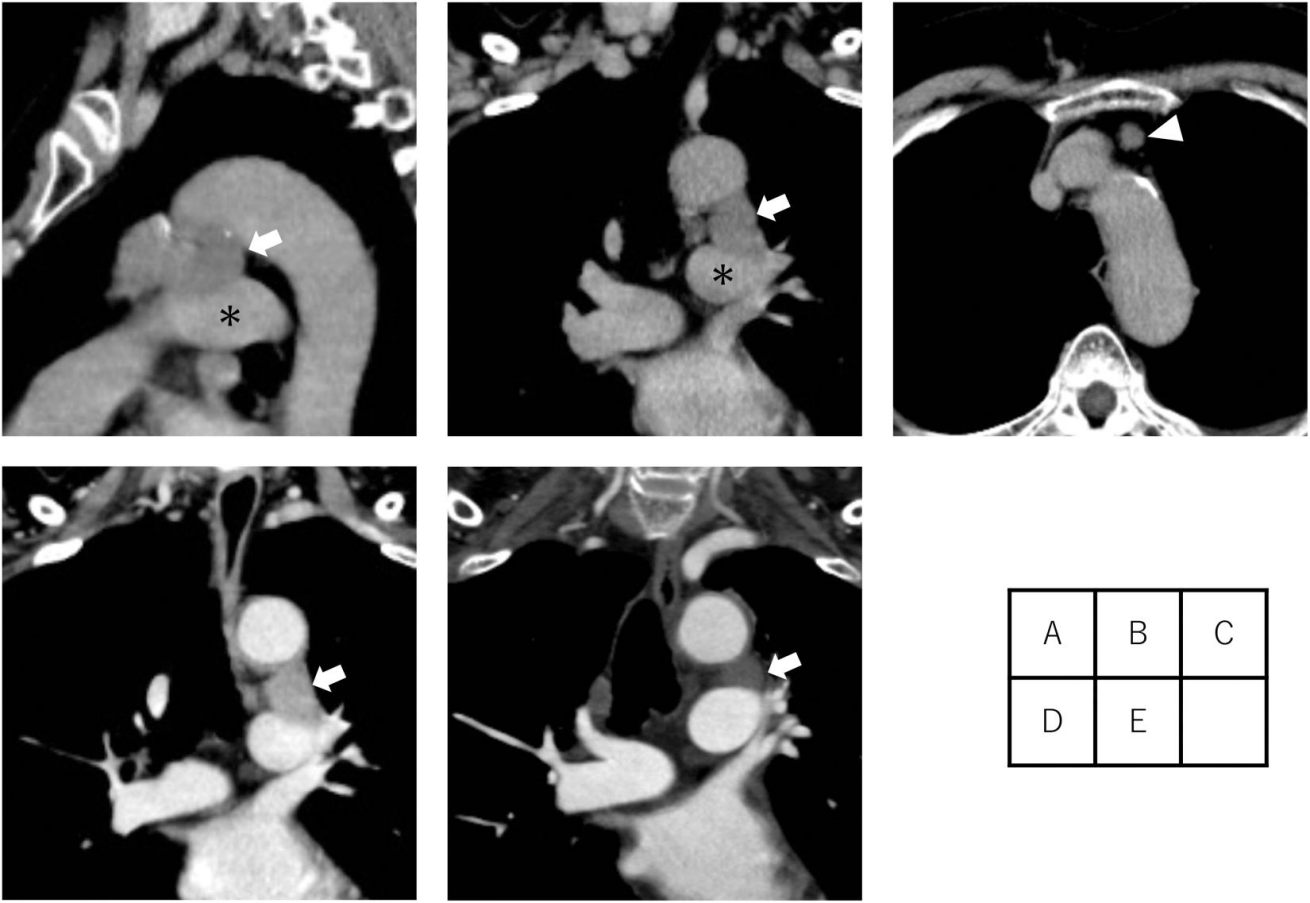
Preoperative CT Findings. (A, B) Anterior mediastinum mass (arrow), which is suspected to have invaded the left main pulmonary artery (asterisk). (C) Enlarged mediastinal lymph nodes (arrowhead). (D) No change in tumour size after chemoradiotherapy (arrow). (E) Tumour reduction after immune checkpoint inhibitor therapy (arrow). Abbreviation: CT, computed tomography.

**Figure 2.**
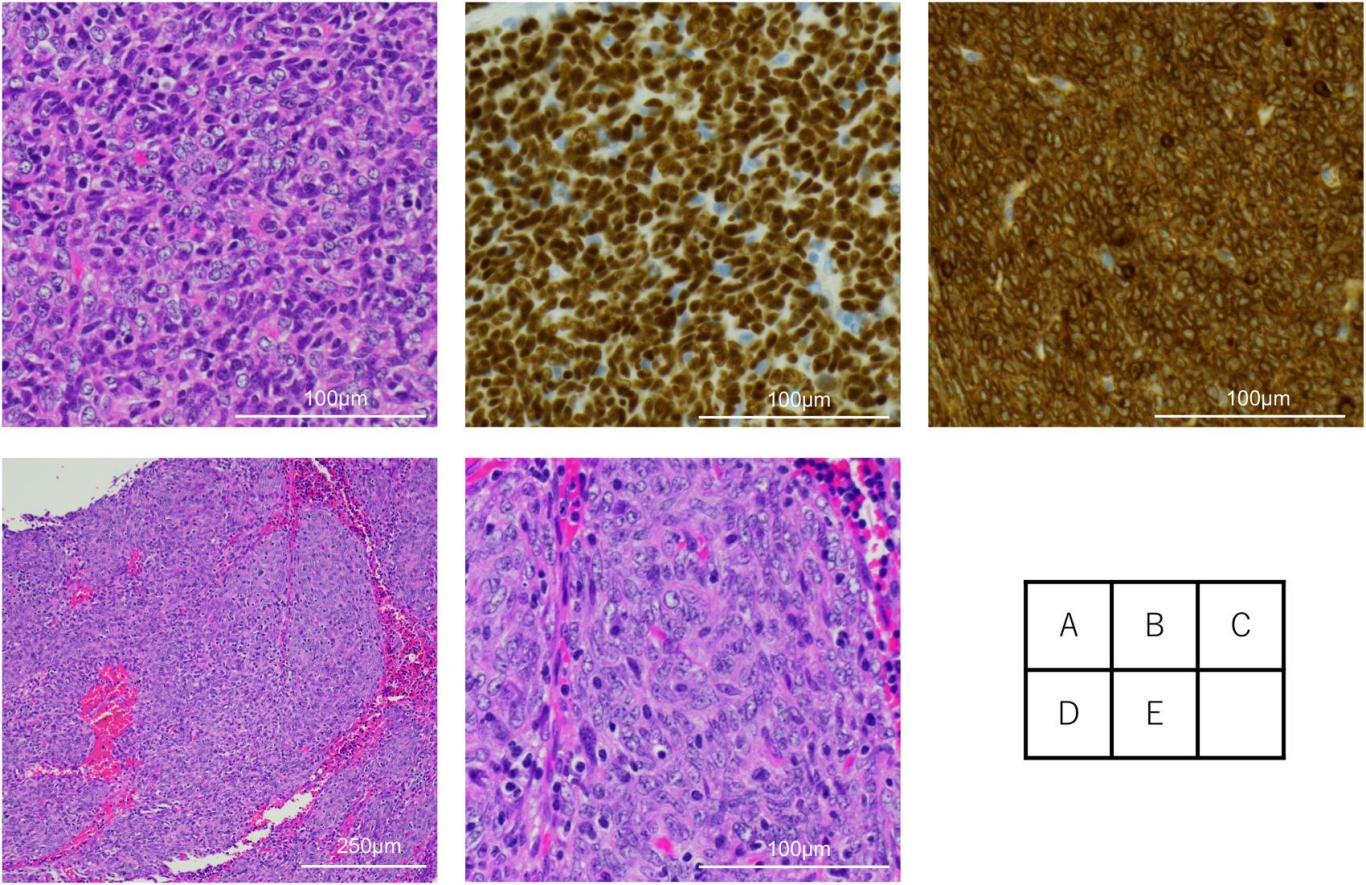
Microscopic Findings. (A) Histopathology of tumour cells in the biopsy showing sheets of atypical polygonal to short spindle cells (H&E), (B and C) immunohistochemically positive for p40 and Bcl-2. (D and E) Residual viable tumour cells are observed in one-third of the resected mediastinal tumour. Abbreviation: H&E, haematoxylin and eosin.

A 67-year-old woman presented with a 4.7 cm anterior mediastinal mass compressing the aortic arch and left main pulmonary artery on chest computed tomography (CT) (Figure 1A and B), along with anterior mediastinal lymph nodes with a short-axis diameter of 1.0 cm (Figure 1C). Positron emission tomography (PET)-CT demonstrated fluorodeoxyglucose uptake in the primary tumour and lymph nodes (SUVmax 2.9 and 3.4, respectively). The tumour was diagnosed as an unresectable thymic epithelial tumour and staged as cStage IVa (T4N1M0, UICC 9th edition), owing to suspected invasion into the intrapericardial left main pulmonary artery. Thoracoscopic biopsy confirmed the diagnosis of thymic squamous cell carcinoma (Figure 2A-C), with high PD-L1 expression (90%-100%).

The patient received definitive concurrent chemoradiotherapy, comprising 6 cycles of weekly carboplatin and paclitaxel with 60 Gy radiation, based on NCCN guidelines. In view of the limited evidence supporting effective treatments for unresectable TC, durvalumab therapy (10 mg/kg every 2 weeks) was subsequently administered following shared decision-making. Written informed consent was obtained. After chemoradiotherapy, no significant reduction in tumour size was observed (Figure 1D). Durvalumab therapy was discontinued after eight cycles due to back pain, later diagnosed as a vertebral compression fracture. One year after the final durvalumab administration, the tumour mass had decreased to 4.2 cm (Figure 1E), and lymphadenopathy had resolved. PET-CT revealed reduced uptake in the primary tumour (SUVmax 2.7), with no residual nodal uptake. Based on the Response Evaluation Criteria in Solid Tumors, the therapeutic response was stable disease with a tumour reduction of 11%. In accordance with the patient’s preference, she remained under close surveillance with CT every 3 months.

Subsequently, salvage surgery was performed via posterolateral thoracotomy 22 months following the final durvalumab administration. Intraoperatively, the tumour was found to have invaded the pericardium and phrenic nerve, necessitating *en bloc* resection of both structures. No intrapericardial great vessel invasion was observed. Dense adhesions and fibrosis surrounding the tumour required sharp dissection from the great vessels to achieve complete resection. The operative time was 285 min, and blood loss was 95 mL. The patient was discharged without complications on postoperative day 9. Histopathology revealed fibrosis in approximately two-thirds of the specimen and residual viable tumour in the remaining portion (Figure 2D and E). The final pathological stage was ypStage II (T2N0M0, UICC 9th edition). Postoperatively, the patient has been followed with CT every 3 months, with no additional treatment. At 4 years and 4 months after initial treatment and 2 years after surgery, the patient remained recurrence-free with no evidence of irAEs.

## DISCUSSION

Treatment options remain extremely limited for unresectable TC, highlighting the need for new therapeutic strategies. Thymic epithelial tumours often exhibit high PD-L1 expression. Several phase II trials of ICI therapy for TC demonstrated clinical efficacy and acceptable tolerability, with better treatment responses observed in patients with high PD-L1 expression. The NCCN guidelines list ICIs as a treatment option for unresectable TC. In the present case, ICI therapy for TC with high PD-L1 expression resulted in tumour size reduction and lymph node downstaging without irAEs.

Complete resection offers a favourable prognosis in patients with TC. However, evidence regarding the role of salvage surgery in TC remains scarce. A retrospective study evaluating the efficacy of salvage surgery for malignant mediastinal tumours reported favourable outcomes, with a 5-year overall survival rate of 88% in 11 patients with thymic epithelial tumours, including 3 patients with TC.^1^ Additionally, four case reports have described successful salvage surgery for initially advanced-stage TC.^2–5^ Among them, a recent report described a complete pathological response after combined radiotherapy and pembrolizumab therapy followed by salvage surgery in a patient with high PD-L1 expression of 85%.^2^ The present case is among the first to demonstrate the potential role of salvage surgery following ICI therapy in select patients with unresectable TC. Further accumulation of clinical experience and evaluation in larger cohorts are warranted to better define patient selection criteria and long-term outcomes.

## Data Availability

The data underlying this article will be shared on reasonable request to the corresponding author.

## References

[1] Petrella F , Leo F, Veronesi G, et al “Salvage” surgery for primary mediastinal malignancies: is it worthwhile? J Thorac Oncol. 2008;3:53-58. 10.1097/JTO.0b013e31815e6d54 18166841

[2] Mendogni P , Orlandi R, Spizzo G, et al Complete pathologic response after concomitant pembrolizumab and radiotherapy in a patient with pretreated metastatic thymic carcinoma: a case report. Mediastinum. 2025;9:21. 10.21037/med-25-16 40666539 PMC12260952

[3] Yamato H , Funaki S, Shimamura K, et al Salvage surgery for stage IVa thymic carcinoma combined with aortic arch resection - case report. J Cardiothorac Surg. 2020;15:305. 10.1186/s13019-020-01354-1 33028405 PMC7542946

[4] Terada J , Toyoda Y, Takeuchi E, et al Surgical resection combined with perioperative chemotherapy for a patient with locally recurrent, previously stage IV thymic small-cell carcinoma: a case report. Thorac Cancer. 2022;13:3415-3419. 10.1111/1759-7714.14717 36345130 PMC9715778

[5] Shimura M , Miura K, Koizumi T, et al Successful resection after first-line lenvatinib therapy in an advanced thymic carcinoma. Thorac Cancer. 2023;14:1640-1643. 10.1111/1759-7714.14913 37132133 PMC10260480

